# *trans*-Bis(1*H*-indole-3-carbaldehyde thio­semicarbazonato-κ^2^ *N* ^1^,*S*)nickel(II)

**DOI:** 10.1107/S1600536808014293

**Published:** 2008-05-17

**Authors:** Mohd. Razali Rizal, Hapipah M. Ali, Seik Weng Ng

**Affiliations:** a Department of Chemistry, University of Malaya, 50603 Kuala Lumpur, Malaysia

## Abstract

The Ni atom in the centrosymmetric title compound, [Ni(C_10_H_9_N_4_S)_2_], is *N*,*S*-chelated by the deprotonated Schiff bases in a square-planar geometry. The –CH=N—N=C(S)—NH_2_ frament is planar. Adjacent mol­ecules are linked by hydrogen bonds between the indolyl –NH (donor) site and the double-bond =N– (acceptor) site of an adjacent mol­ecule, forming a layer motif.

## Related literature

For the structure of the neutral Schiff base, see: Rizal *et al.* (2008 ▶). For background literature on the medicinal activity of metal complexes of the Schiff base and related compounds, see: Husain *et al.* (2007 ▶); Wilson *et al.* (2005 ▶).
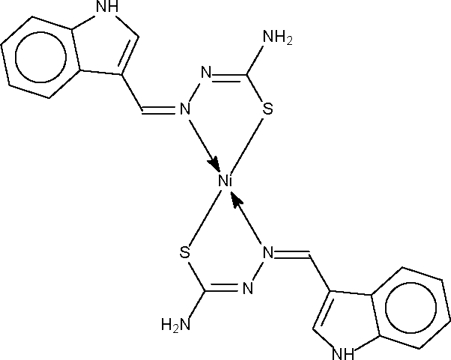


## Experimental

### 

#### Crystal data

[Ni(C_10_H_9_N_4_S)_2_]
*M* *_r_* = 493.25
Monoclinic, 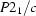

*a* = 10.4388 (3) Å
*b* = 5.2604 (1) Å
*c* = 19.1122 (5) Å
β = 104.803 (2)°
*V* = 1014.66 (4) Å^3^
*Z* = 2
Mo *K*α radiation
μ = 1.19 mm^−1^
*T* = 100 (2) K
0.14 × 0.04 × 0.01 mm

#### Data collection

Bruker SMART APEX diffractometer
Absorption correction: multi-scan (*SADABS*; Sheldrick, 1996 ▶) *T*_min_ = 0.851, *T*_max_ = 0.988
12357 measured reflections
2326 independent reflections
1774 reflections with *I* > 2σ(*I*)
*R*_int_ = 0.062

#### Refinement

*R*[*F* ^2^ > 2σ(*F* ^2^)] = 0.034
*wR*(*F* ^2^) = 0.081
*S* = 1.02
2326 reflections
154 parameters
3 restraints
H atoms treated by a mixture of independent and constrained refinement
Δρ_max_ = 0.43 e Å^−3^
Δρ_min_ = −0.30 e Å^−3^

### 

Data collection: *APEX2* (Bruker, 2007 ▶); cell refinement: *SAINT* (Bruker, 2007 ▶); data reduction: *SAINT*; program(s) used to solve structure: *SHELXS97* (Sheldrick, 2008 ▶); program(s) used to refine structure: *SHELXL97* (Sheldrick, 2008 ▶); molecular graphics: *X-SEED* (Barbour, 2001 ▶; Dolomanov *et al.*, 2003); software used to prepare material for publication: *publCIF* (Westrip, 2008 ▶).

## Supplementary Material

Crystal structure: contains datablocks global, I. DOI: 10.1107/S1600536808014293/sg2241sup1.cif

Structure factors: contains datablocks I. DOI: 10.1107/S1600536808014293/sg2241Isup2.hkl

Additional supplementary materials:  crystallographic information; 3D view; checkCIF report

## Figures and Tables

**Table:** 

Ni1—N2	1.918 (2)
Ni1—S1	2.1669 (6)

**Table:** 

N2—Ni1—S1	85.72 (6)
N2—Ni1—S1^i^	94.28 (6)

Symmetry code: (i) 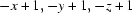.

**Table 2:** Hydrogen-bond geometry (Å, °)

*D*—H⋯*A*	*D*—H	H⋯*A*	*D*⋯*A*	*D*—H⋯*A*
N1—H1n⋯N3^ii^	0.88 (3)	2.06 (2)	2.876 (3)	155 (3)

Symmetry code: (ii) 
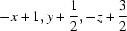
.

**Fig. 1.**
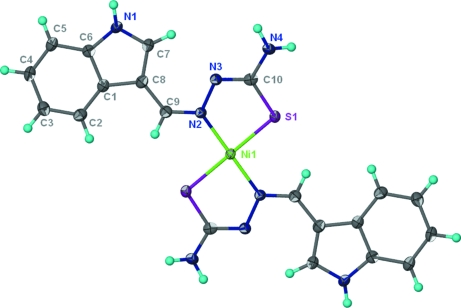
Thermal ellipsoid plot of Ni(C10H9N4S)2 at the 70% probability level; hydrogen atoms are drawn as spheres of arbitrary radius. The molecule lies on a center-of-inversion. Unlabeled atoms are related to the labeled ones by this symmetry element.

**Fig. 2.**
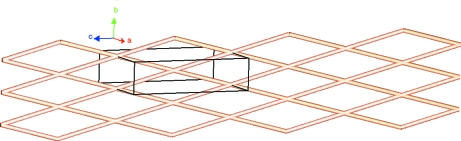
OLEX (Dolomanov et al., 2003) representation of the hydrogen-bonded layer motif.

## supplementary crystallographic information

### Comment

A previous study reports the structure of 1*H*-indole-3-carboxaldehyde thiosemicarbazone (Rizal *et al.*, 2008). The compound in its deprotonated form can function as a bidentate chelate, and this is confirmed in the present nickel(II) derivative (Scheme I, Fig. 1). The metal center lies on a center-of-inversion in a square planar coordination geometry. Adjacent molecules are linked by hydrogen bonds between the indolyl –NH (donor) site and the double-bond =N– (acceptor) site of an adjacent molecule to form a layer motif (Fig. 2).

### Experimental

Nickel acetate tetrahydrate (0.06 g,0.22 mmol) and 1*H*-indole-3-carboxaldehyde thiosemicarbazone (0.10 g, 0.44 mmol), ethanol (4 ml) and water (10 ml) were sealed in a 15-ml, Teflon-lined, Parr bomb. The bomb was heated at 383 K for 2 days. The bomb when cooled to room temperature over a day to give orange plates.

### Refinement

Carbon-bound H-atoms were placed in calculated positions (C—H 0.95 Å) and were included in the refinement in the riding model approximation, with *U*(H) set to 1.2*U*(C). The nitrogen-bound H-atoms were located in a difference Fourier map, and were refined with an N–H distance restraint of 0.88±0.01 Å; their temperature factors were freely refined.

### Crystal data

**Table:**

[Ni(C_10_H_9_N_4_S)_2_]	*F*_000_ = 508
*M**_r_* = 493.25	*D*_x_ = 1.614 Mg m^−^^3^
Monoclinic, *P*2_1_/*c*	Mo *K*α radiation λ = 0.71073 Å
Hall symbol: -P 2ybc	Cell parameters from 1799 reflections
*a* = 10.4388 (3) Å	θ = 2.6–24.7º
*b* = 5.2604 (1) Å	µ = 1.19 mm^−^^1^
*c* = 19.1122 (5) Å	*T* = 100 (2) K
β = 104.803 (2)º	Plate, orange
*V* = 1014.66 (4) Å^3^	0.14 × 0.04 × 0.01 mm
*Z* = 2	

### Data collection

**Table:**

Bruker SMART APEX diffractometer	2326 independent reflections
Radiation source: fine-focus sealed tube	1774 reflections with *I* > 2σ(*I*)
Monochromator: graphite	*R*_int_ = 0.062
*T* = 100(2) K	θ_max_ = 27.5º
φ and ω scans	θ_min_ = 2.0º
Absorption correction: Multi-scan(SADABS; Sheldrick, 1996)	*h* = −12→13
*T*_min_ = 0.851, *T*_max_ = 0.988	*k* = −6→6
12357 measured reflections	*l* = −24→24

### Refinement

**Table:**

Refinement on *F*^2^	Secondary atom site location: difference Fourier map
Least-squares matrix: full	Hydrogen site location: inferred from neighbouring sites
*R*[*F*^2^ > 2σ(*F*^2^)] = 0.034	H atoms treated by a mixture of independent and constrained refinement
*wR*(*F*^2^) = 0.081	*w* = 1/[σ^2^(*F*_o_^2^) + (0.0362*P*)^2^ + 0.5143*P*] where *P* = (*F*_o_^2^ + 2*F*_c_^2^)/3
*S* = 1.02	(Δ/σ)_max_ = 0.001
2326 reflections	Δρ_max_ = 0.43 e Å^−^^3^
154 parameters	Δρ_min_ = −0.30 e Å^−^^3^
3 restraints	Extinction correction: none
Primary atom site location: structure-invariant direct methods	

### Fractional atomic coordinates and isotropic or equivalent isotropic displacement parameters (Å^2^)

**Table:**

	*x*	*y*	*z*	*U*_iso_*/*U*_eq_	
Ni1	0.5000	0.5000	0.5000	0.01261 (12)	
S1	0.33444 (6)	0.74950 (12)	0.45463 (3)	0.01747 (15)	
N1	0.6654 (2)	1.1528 (4)	0.78929 (11)	0.0171 (5)	
H1N	0.637 (3)	1.262 (5)	0.8165 (14)	0.045 (10)*	
N2	0.52205 (19)	0.6972 (4)	0.58664 (10)	0.0143 (4)	
N3	0.42554 (19)	0.8723 (4)	0.59419 (10)	0.0153 (4)	
N4	0.2345 (2)	1.0700 (4)	0.53342 (12)	0.0205 (5)	
H4N1	0.240 (3)	1.177 (5)	0.5691 (12)	0.042 (10)*	
H4N2	0.184 (3)	1.121 (6)	0.4919 (10)	0.043 (10)*	
C1	0.7786 (2)	0.8186 (5)	0.76257 (12)	0.0154 (5)	
C2	0.8845 (2)	0.6471 (5)	0.77478 (13)	0.0182 (5)	
H2	0.8869	0.5159	0.7410	0.022*	
C3	0.9859 (2)	0.6731 (5)	0.83732 (13)	0.0195 (5)	
H3	1.0588	0.5590	0.8460	0.023*	
C4	0.9830 (2)	0.8646 (5)	0.88809 (13)	0.0190 (5)	
H4	1.0538	0.8766	0.9306	0.023*	
C5	0.8795 (2)	1.0361 (5)	0.87760 (12)	0.0178 (5)	
H5	0.8774	1.1659	0.9118	0.021*	
C6	0.7782 (2)	1.0092 (5)	0.81421 (12)	0.0159 (5)	
C7	0.5945 (2)	1.0621 (5)	0.72458 (12)	0.0166 (5)	
H7	0.5132	1.1309	0.6969	0.020*	
C8	0.6586 (2)	0.8537 (5)	0.70493 (12)	0.0166 (5)	
C9	0.6276 (2)	0.6972 (5)	0.64112 (12)	0.0163 (5)	
H9	0.6935	0.5761	0.6381	0.020*	
C10	0.3354 (2)	0.9073 (5)	0.53374 (13)	0.0159 (5)	

### Atomic displacement parameters (Å^2^)

**Table:**

	*U*^11^	*U*^22^	*U*^33^	*U*^12^	*U*^13^	*U*^23^
Ni1	0.0129 (2)	0.0143 (2)	0.0104 (2)	0.00022 (19)	0.00260 (16)	−0.00047 (18)
S1	0.0180 (3)	0.0207 (3)	0.0124 (3)	0.0042 (3)	0.0014 (2)	−0.0010 (2)
N1	0.0177 (11)	0.0189 (11)	0.0140 (10)	0.0007 (9)	0.0029 (8)	−0.0039 (9)
N2	0.0161 (10)	0.0141 (10)	0.0127 (9)	0.0014 (8)	0.0036 (8)	−0.0004 (8)
N3	0.0155 (11)	0.0171 (11)	0.0137 (10)	0.0021 (9)	0.0047 (8)	−0.0002 (8)
N4	0.0225 (12)	0.0210 (12)	0.0172 (11)	0.0080 (9)	0.0035 (9)	−0.0025 (9)
C1	0.0156 (12)	0.0156 (12)	0.0151 (11)	−0.0034 (10)	0.0042 (10)	0.0002 (9)
C2	0.0190 (13)	0.0192 (13)	0.0177 (12)	−0.0019 (10)	0.0068 (10)	−0.0025 (10)
C3	0.0153 (13)	0.0225 (14)	0.0205 (12)	0.0005 (11)	0.0040 (10)	0.0028 (11)
C4	0.0166 (13)	0.0245 (14)	0.0148 (11)	−0.0037 (11)	0.0018 (10)	−0.0005 (10)
C5	0.0196 (13)	0.0207 (14)	0.0127 (11)	−0.0040 (11)	0.0034 (10)	−0.0005 (10)
C6	0.0172 (12)	0.0167 (12)	0.0151 (11)	−0.0011 (11)	0.0063 (9)	0.0013 (10)
C7	0.0166 (12)	0.0190 (14)	0.0137 (11)	−0.0016 (10)	0.0030 (10)	−0.0003 (9)
C8	0.0191 (13)	0.0177 (13)	0.0135 (11)	−0.0022 (10)	0.0051 (10)	0.0001 (10)
C9	0.0175 (12)	0.0176 (13)	0.0145 (11)	0.0008 (10)	0.0055 (10)	0.0003 (10)
C10	0.0180 (13)	0.0140 (12)	0.0182 (12)	−0.0033 (10)	0.0092 (10)	0.0007 (10)

### Geometric parameters (Å, °)

**Table:**

Ni1—N2^i^	1.919 (2)	C1—C6	1.408 (3)
Ni1—N2	1.918 (2)	C1—C8	1.453 (3)
Ni1—S1^i^	2.1669 (6)	C2—C3	1.386 (3)
Ni1—S1	2.1669 (6)	C2—H2	0.9500
S1—C10	1.723 (2)	C3—C4	1.404 (4)
N1—C7	1.355 (3)	C3—H3	0.9500
N1—C6	1.377 (3)	C4—C5	1.382 (4)
N1—H1n	0.88 (3)	C4—H4	0.9500
N2—C9	1.309 (3)	C5—C6	1.397 (3)
N2—N3	1.399 (3)	C5—H5	0.9500
N3—C10	1.303 (3)	C7—C8	1.385 (3)
N4—C10	1.355 (3)	C7—H7	0.9500
N4—H4n1	0.88 (3)	C8—C9	1.438 (3)
N4—H4n2	0.88 (3)	C9—H9	0.9500
C1—C2	1.400 (3)		
			
N2^i^—Ni1—N2	180.000 (1)	C2—C3—H3	119.3
N2^i^—Ni1—S1^i^	85.72 (6)	C4—C3—H3	119.3
N2—Ni1—S1	85.72 (6)	C5—C4—C3	121.5 (2)
N2—Ni1—S1^i^	94.28 (6)	C5—C4—H4	119.3
N2^i^—Ni1—S1	94.28 (6)	C3—C4—H4	119.3
S1^i^—Ni1—S1	180.0	C4—C5—C6	116.8 (2)
C10—S1—Ni1	96.63 (9)	C4—C5—H5	121.6
C7—N1—C6	110.0 (2)	C6—C5—H5	121.6
C7—N1—H1N	126 (2)	N1—C6—C5	129.5 (2)
C6—N1—H1N	123 (2)	N1—C6—C1	107.7 (2)
C9—N2—N3	113.60 (19)	C5—C6—C1	122.9 (2)
C9—N2—Ni1	125.30 (17)	N1—C7—C8	109.7 (2)
N3—N2—Ni1	120.96 (14)	N1—C7—H7	125.1
C10—N3—N2	112.16 (19)	C8—C7—H7	125.1
C10—N4—H4N1	121 (2)	C7—C8—C9	131.6 (2)
C10—N4—H4N2	119 (2)	C7—C8—C1	106.1 (2)
H4N1—N4—H4N2	114 (3)	C9—C8—C1	122.2 (2)
C2—C1—C6	119.1 (2)	N2—C9—C8	129.5 (2)
C2—C1—C8	134.4 (2)	N2—C9—H9	115.3
C6—C1—C8	106.5 (2)	C8—C9—H9	115.3
C3—C2—C1	118.5 (2)	N3—C10—N4	118.5 (2)
C3—C2—H2	120.8	N3—C10—S1	123.44 (19)
C1—C2—H2	120.8	N4—C10—S1	118.03 (18)
C2—C3—C4	121.3 (2)		
			
N2^i^—Ni1—S1—C10	172.73 (10)	C8—C1—C6—N1	−0.4 (3)
N2—Ni1—S1—C10	−7.27 (10)	C2—C1—C6—C5	0.1 (4)
S1^i^—Ni1—N2—C9	15.3 (2)	C8—C1—C6—C5	−179.6 (2)
S1—Ni1—N2—C9	−164.7 (2)	C6—N1—C7—C8	0.3 (3)
S1^i^—Ni1—N2—N3	−169.40 (16)	N1—C7—C8—C9	−177.4 (2)
S1—Ni1—N2—N3	10.60 (16)	N1—C7—C8—C1	−0.5 (3)
C9—N2—N3—C10	166.4 (2)	C2—C1—C8—C7	−179.1 (3)
Ni1—N2—N3—C10	−9.4 (3)	C6—C1—C8—C7	0.6 (3)
C6—C1—C2—C3	−0.5 (4)	C2—C1—C8—C9	−1.8 (4)
C8—C1—C2—C3	179.2 (3)	C6—C1—C8—C9	177.8 (2)
C1—C2—C3—C4	0.6 (4)	N3—N2—C9—C8	−2.0 (4)
C2—C3—C4—C5	−0.4 (4)	Ni1—N2—C9—C8	173.7 (2)
C3—C4—C5—C6	0.1 (4)	C7—C8—C9—N2	−7.0 (5)
C7—N1—C6—C5	179.2 (2)	C1—C8—C9—N2	176.5 (2)
C7—N1—C6—C1	0.1 (3)	N2—N3—C10—N4	179.1 (2)
C4—C5—C6—N1	−178.9 (2)	N2—N3—C10—S1	1.4 (3)
C4—C5—C6—C1	0.1 (4)	Ni1—S1—C10—N3	5.4 (2)
C2—C1—C6—N1	179.3 (2)	Ni1—S1—C10—N4	−172.37 (19)

Symmetry codes: (i) −*x*+1, −*y*+1, −*z*+1.

### Hydrogen-bond geometry (Å, °)

**Table:**

*D*—H···*A*	*D*—H	H···*A*	*D*···*A*	*D*—H···*A*
N1—H1n···N3^ii^	0.88 (3)	2.06 (2)	2.876 (3)	155 (3)

Symmetry codes: (ii) −*x*+1, *y*+1/2, −*z*+3/2.
